# Association between disease awareness and prognosis in patients with glioblastoma: A retrospective cohort study

**DOI:** 10.1097/MD.0000000000050163

**Published:** 2026-08-28

**Authors:** Yanjiao Wu, Xiaoying Xue, Shuying Ji, Wenyan Wang, Lei Tian, Xuetao Han, Linlin Su, Junling Liu

**Affiliations:** a Department of Radiotherapy, The Second Hospital of Hebei Medical University, Shijiazhuang, China.

**Keywords:** disease awareness, glioblastoma, overall survival, prognosis

## Abstract

This study aimed to evaluate whether disease awareness among Chinese patients with glioblastoma was associated with prognosis. This single-center, retrospective cohort study included consecutive patients diagnosed with glioblastoma between February 2009 and August 2020. Disease awareness was categorized as full awareness, midway awareness, awareness of lesser severity, and unawareness based on documented medical records and follow-up information. Overall survival (OS) and recurrence-free survival (RFS) were analyzed using Kaplan–Meier and Cox regression methods, with censoring applied for patients without death or recurrence events. Ninety-three patients were included. The median age was 53 years (interquartile range [IQR], 45–64), and 57.0% were male. The patients were classified according to disease awareness category: full awareness in 25.8%, midway awareness in 26.9%, awareness of lesser severity in 21.5%, and unawareness in 25.8%. After a median follow-up of 37.81 months, the median OS was 21.11 months (95% confidence interval [CI]: 19.30–32.98), and the median RFS was 24.99 months (95% CI: 16.01-not estimable). Disease awareness was not significantly associated with OS or RFS after adjustment for potential confounders, including treatments. Midway awareness showed a nonsignificant numerical pattern toward longer OS (hazard ratio, 0.631; 95% CI: 0.331–1.204; *P* = .163). Disease awareness level was not significantly associated with OS or RFS in this cohort. The numerically longer OS observed among patients with midway awareness should be interpreted as exploratory and hypothesis-generating and requires confirmation in larger prospective studies.

## 1. Introduction

Glioblastoma is an aggressive, malignant primary brain tumor deriving from glial cells (including oligodendrocytes, astrocytes, and ependymal cells) or their precursors.^[1,2]^ The only environmental exposure known to be associated with glioblastoma is ionizing radiation exposure,^[3]^ while genetic risk factors include Li Fraumeni syndrome, neurofibromatosis type 1, Cowden disease, and von Hippel–Lindau syndrome.^[1,4]^ The annual age-adjusted incidence of glioblastoma in the United States of America is 3.27 per 100,000 persons.^[5]^ In China, glioblastoma represents 8.56% of all brain tumors.^[6]^ Glioblastoma is associated with a poor prognosis; for example, data from the United States show a 5-year survival rate of merely 7%.^[1]^ Similarly, recent data from the National Brain Tumour Registry of China indicate a poor prognosis for Chinese patients, with a reported median overall survival of 17.6 months.^[6]^ Such poor prognosis is related to the limited efficacy of available treatments. Gross total resection can help alleviate symptoms and improve prognosis, but recurrence is inevitable.^[7]^ Radiotherapy with concurrent and adjuvant temozolomide is the standard recommended treatment for glioblastoma,^[8]^ but the survival rate at 5 years was also limited at about 10% (Table 1).^[9]^

**Table 1:** Baseline demographic and clinical characteristics and distribution of disease awareness categories in the cohort.

Characteristic	Total (n = 93)
Age, years, median (IQR)	53.00 (45.00, 64.00)
Age group, n (%)	
≤50 yr	37 (39.8)
>50 yr	56 (60.2)
Gender, n (%)	
Female	40 (43.0)
Male	53 (57.0)
Pathology, n (%)	
Glioblastoma	93 (100.0)
Apatinib treatment, n (%)	
No	76 (81.7)
Yes	17 (18.3)
Bevacizumab treatment, n (%)	
No	77 (82.8)
Yes	16 (17.2)
Disease awareness category, n (%)	
Full awareness	24 (25.8)
Midway awareness	25 (26.9)
Awareness of lesser severity	20 (21.5)
Unawareness	24 (25.8)

Values are presented as n (%) or median (interquartile range), as appropriate. Treatment variables indicate whether the patient received each therapy and are not mutually exclusive; therefore, percentages for apatinib and bevacizumab are not expected to total 100%.

IQR = interquartile range.

In Chinese society, it is not uncommon for physicians and/or family members to withhold the full extent of a patient’s medical condition (e.g., cancer diagnosis, disease stage, or life expectancy) from the patient. This is often related to traditional beliefs, family preferences, and physicians’ views on information disclosure.^[10-12]^ A good understanding of prognosis may help patients prepare advance directives, have necessary end-of-life conversations with loved ones, and address end-of-life concerns.^[13]^ In terminally ill patients or patients with very aggressive cancers, prognostic awareness can influence end-of-life decisions.^[14]^ Inaccurate prognostic awareness can have varying impacts on treatment decisions and prognosis.^[14]^ Nevertheless, clinical practice also aims to minimize additional psychological burden and preserve treatment adherence in critical diseases.^[15]^ Patient awareness of the disease is related to quality of life, since patients may need to make decisions regarding cancer treatments and weigh quality of life against length of life.^[16]^ Limited or partial awareness of prognosis may, in some situations, function as a psychological defense mechanism by reducing anxiety, fear, and emotional turmoil.^[17,18]^ Conversely, studies in cancer populations have suggested that greater patient awareness of diagnosis, treatment options, and potential side effects may be associated with treatment adherence, patient engagement, and clinical outcomes.^[19-23]^

As a highly aggressive malignant tumor, glioblastoma occurrence and progression may be influenced by psychological factors.^[24]^ Stress, anxiety, and depression have been recognized as factors that may decrease survival in patients with primary brain tumors.^[24]^ Emotional fluctuations, especially chronic stress, can exacerbate brain tumor progression.^[25,26]^ Hence, patients’ awareness of their condition may affect psychological health and quality of life, which could in turn influence cancer prognosis.^[27,28]^ Among patients with glioblastoma, illness awareness may be closely related to prognostic understanding, communication, and cognitive function.^[29]^ When patients are informed of the true condition and prognosis of glioblastoma, they may experience varying emotional reactions that could affect treatment adherence and overall quality of life.^[30]^ While general prognostic data for glioblastoma in China exist, evidence is lacking on whether different levels of disease awareness are associated with prognosis in Chinese patients with glioblastoma. This gap provides the rationale for the present study.

Therefore, this study aimed to investigate whether the awareness of illness among patients with glioblastoma in China was associated with prognosis. The results may provide preliminary evidence to inform future physician-patient-family communication and psychological support, without implying a specific disclosure strategy.

## 2. Materials and methods

### 2.1. Study design and patients

This single-center, retrospective cohort study was conducted at The Second Hospital of Hebei Medical University and included consecutive patients diagnosed with and treated for glioblastoma between February 2009 and August 2020. Data on disease awareness and prognosis were obtained from medical charts, including admission records, progress notes, discharge summaries, outpatient follow-up records, family communication notes when available, and telephone follow-up records.

The study adhered to the principles of the Declaration of Helsinki and was approved by the ethics committee of The Second Hospital of Hebei Medical University (approval No.: 2025-R585). The requirement for individual informed consent was waived by the ethics committee because of the retrospective nature of the study.

The inclusion criteria were patients aged 6 years or older, irrespective of gender; pathologically confirmed diagnosis of glioblastoma; and at least 1 follow-up record to evaluate survival status. The exclusion criteria were lacking critical clinical information (e.g., pathological data or treatment records); other malignancies or severe illnesses that could significantly affect the survival outcomes; or patients whose awareness of their condition could not be accurately determined.

The patients received treatments as per routine clinical practice. These included surgical interventions (irrespective of the resection extent intended) and systemic targeted therapies like bevacizumab or apatinib for patients with postoperative residual disease or those ineligible for surgery.

### 2.2. Data collection

The data were retrospectively gathered and included demographic characteristics (e.g., age and gender), treatment details (e.g., surgery and targeted therapies), follow-up status, and patient awareness status. Disease awareness was determined from documented medical record entries and follow-up information indicating what the patient had been told about the diagnosis, severity, and prognosis. Patients were assigned to one of 4 categories: full awareness, defined as documentation that the patient knew the diagnosis of glioblastoma or malignant brain tumor and its serious prognosis from the time of diagnosis; midway awareness, defined as documentation that the patient was initially unaware of the true diagnosis or severity but was later informed of the true diagnosis and/or severity during the treatment or follow-up course; awareness of lesser severity, defined as documentation that the patient knew they had a brain tumor or related condition but was told a less severe explanation, such as a lower-grade or nonmalignant condition, often according to family preference; and unawareness, defined as documentation that the patient was not informed of the diagnosis or its severity during the recorded treatment and follow-up period, with family members acting as the main recipients of disease information. Patients whose awareness category could not be determined from the available records were excluded. The time points for each follow-up, survival status (alive/deceased), and disease status (recurrence/no recurrence) were recorded. Disease recurrence was assessed based on imaging findings and radiologists’ reports.

### 2.3. Study outcomes

The primary outcome was overall survival (OS), defined as the time from diagnosis to death from any cause. Patients who were alive at the last follow-up were censored at the date of last contact. The secondary outcome was recurrence-free survival (RFS), defined as the time from diagnosis to radiologically documented recurrence. Patients without documented recurrence were censored at the last disease assessment or last follow-up, as applicable.

### 2.4. Statistical analysis

Descriptive statistics were used to summarize the patient characteristics. Categorical variables were reported as n (%). The continuous variables were tested for normality using the Shapiro–Wilk test. Continuous variables following a normal distribution were presented as means ± standard deviations, while those not following a normal distribution were presented as medians (interquartile ranges). The median survival time and 95% confidence intervals (CIs) for OS and RFS were estimated using the Kaplan–Meier method, with group comparisons performed using the log-rank test. The factors influencing the outcomes were analyzed using univariable and multivariable Cox regression analyses. Variables with a *P*-value < .1 in the univariable analyses were included in the multivariable analysis. All tests were 2-sided, and *P*-values < .05 were considered statistically significant. All analyses were conducted using R 4.4.0 (The R Project for Statistical Computing, www.r-project.org).

## 3. Results

### 3.1. Characteristics and awareness status of the patients

Ninety-three patients were included. The median age was 53 (interquartile range, 45–64) years, and 57.0% were male. All patients were confirmed to have glioblastoma. Apatinib was administered to 18.3% and bevacizumab to 17.2% of patients. These systemic therapies were not mutually exclusive; therefore, treatment percentages were not expected to add up to 100%. The patients were categorized according to disease awareness category: full awareness in 25.8%, midway awareness in 26.9%, awareness of lesser severity in 21.5%, and unawareness in 25.8%.

### 3.2. Patient prognosis

**Figure 1.**
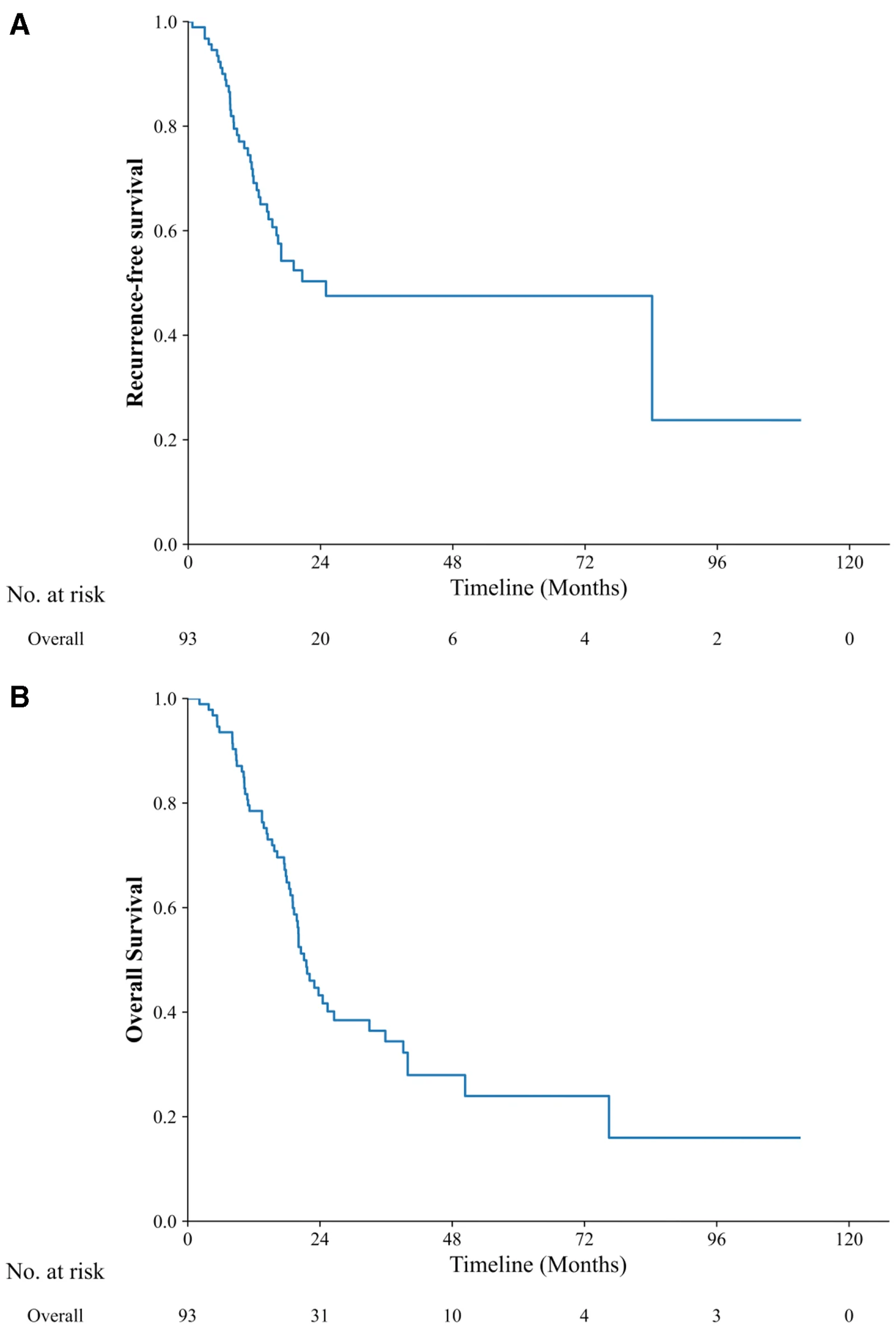
Kaplan–Meier curves for survival outcomes in the overall cohort. (A) Recurrence-free survival (RFS), defined as the time from diagnosis to radiologically documented recurrence; patients without documented recurrence were censored at the last disease assessment or follow-up. (B) Overall survival (OS), defined as the time from diagnosis to death from any cause; patients alive at last contact were censored at last follow-up. The *x*-axis shows time from diagnosis in months, and the *y*-axis shows estimated survival probability. OS = overall survival, RFS = recurrence-free survival.

The median follow-up time was 37.81 months (range, 2.14–110.99). Forty patients (43.0%) had a documented recurrence event; patients without recurrence were censored as described in the Methods. The median RFS was 24.99 (95% confidence interval [CI]: 16.01-not estimable) months (Table 2 and Fig. 1A). Among the 93 patients, 59 (63.4%) died; patients alive at last contact were censored. The median OS was 21.11 (95% CI: 19.30–32.98) months (Table 2 and Fig. 1B).

**Table 2:** Follow-up, recurrence-free survival, and overall survival in the cohort.

Outcome	Total (n = 93)
Recurrence, n (%)	40 (43.0)
RFS, months, median (95% CI)	24.99 (16.01–not estimable)
Death, n (%)	59 (63.4)
OS, months, median (95% CI)	21.11 (19.30–32.98)
Median follow-up, months, median (range)	37.81 (2.14–110.99)

Overall survival was defined as the time from diagnosis to death from any cause, with patients alive at last follow-up censored at last contact. Recurrence-free survival was defined as the time from diagnosis to radiologically documented recurrence, with patients without recurrence censored at the last disease assessment or follow-up. Not estimable indicates that the upper confidence limit was not reached.

CI = confidence interval, OS = overall survival, RFS = recurrence-free survival.

### 3.3. Prognostic differences among subgroups

**Figure 2.**
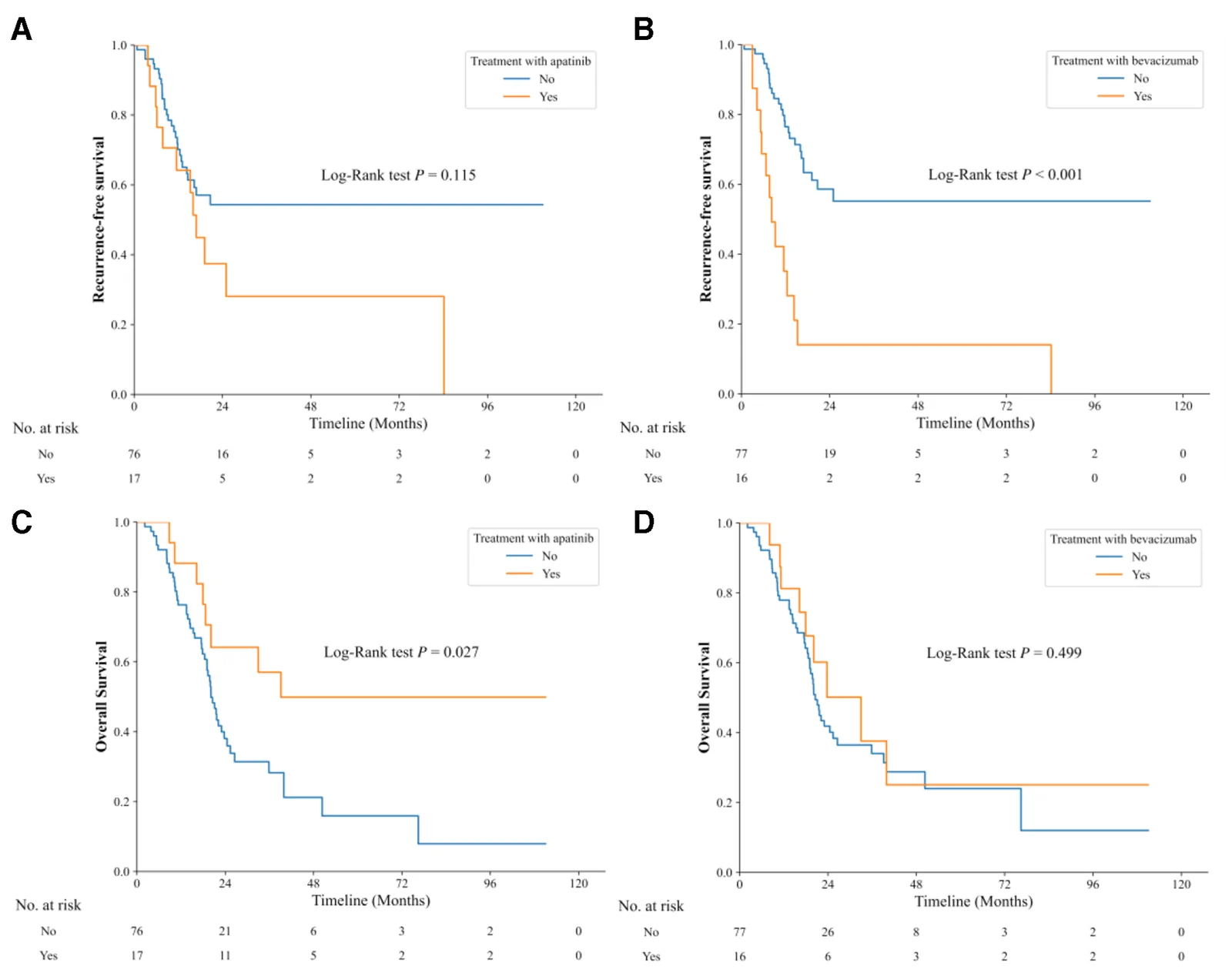
Kaplan–Meier subgroup analyses of recurrence-free survival and overall survival according to targeted therapy exposure. (A) RFS according to apatinib treatment. (B) RFS according to bevacizumab treatment. (C) OS according to apatinib treatment. (D) OS according to bevacizumab treatment. Apatinib and bevacizumab categories were not mutually exclusive. The *x*-axis shows time from diagnosis in months, and the *y*-axis shows estimated recurrence-free or overall survival probability. *P*-values were calculated using the log-rank test. OS = overall survival, RFS = recurrence-free survival.

**Figure 3.**
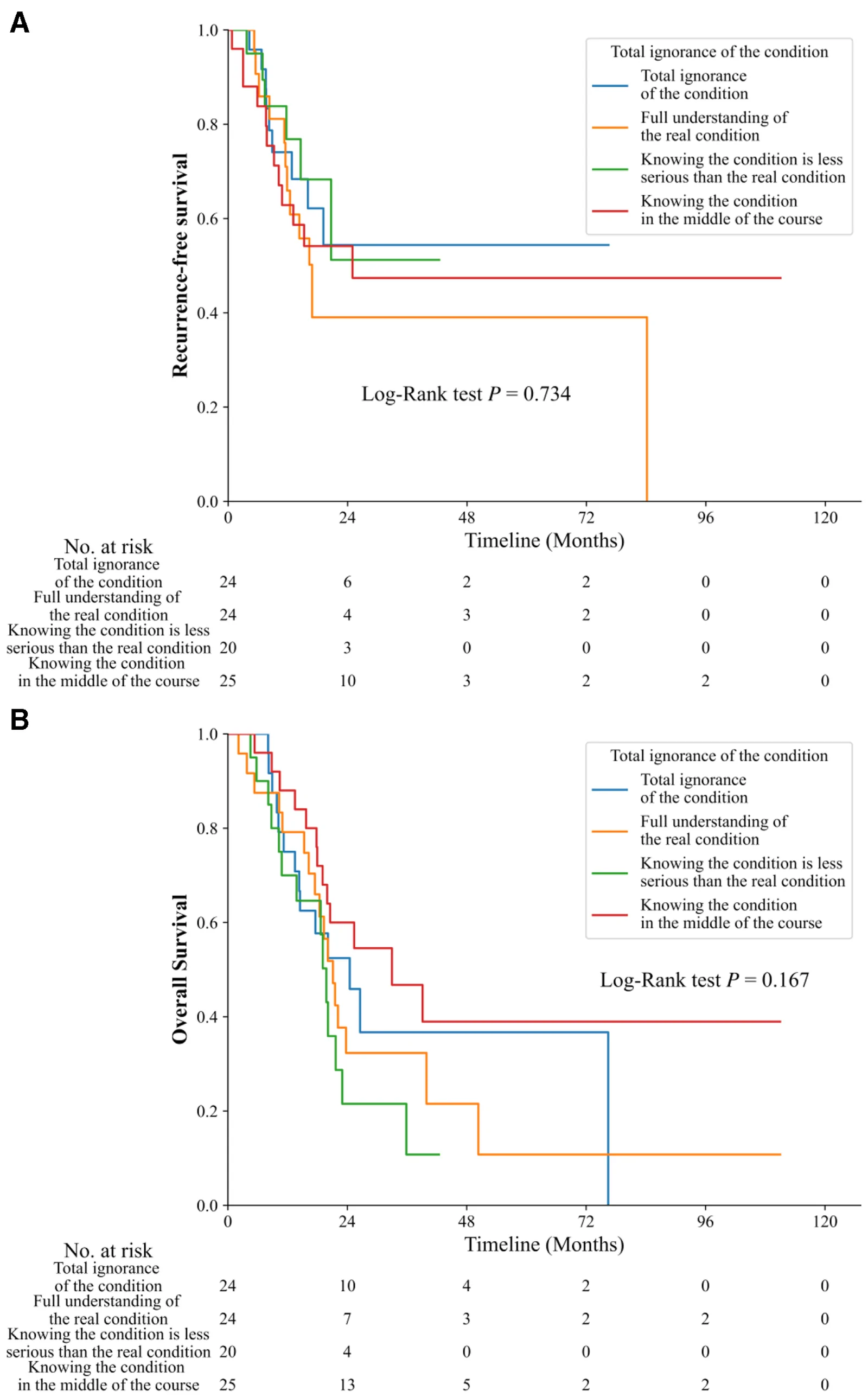
Kaplan–Meier subgroup analyses of survival outcomes according to disease awareness category. (A) Recurrence-free survival and (B) overall survival across 4 disease awareness categories: full awareness, midway awareness, awareness of lesser severity, and unawareness. The *x*-axis shows time from diagnosis in months, and the *y*-axis shows estimated survival probability. *P*-values were calculated using the log-rank test. Group definitions are provided in the Methods.

As shown in Table 3 and Figure 2, apatinib was not associated with RFS (Fig. 2A), and bevacizumab was associated with a shorter median RFS (8.22 vs not estimable, *P* < .001; Fig. 2B). However, apatinib treatment was associated with a longer median OS (39.12 vs 20.12 months, *P* = .027; Fig. 2C), and bevacizumab was not associated with OS (Fig. 2D). The other clinical variables (e.g., gender and age) were not associated with OS or RFS (Table 3). For the disease awareness categories, there were no statistically significant differences in RFS (Fig. 3A) or OS (Fig. 3B). The midway awareness group had a numerically longer median OS (33.00 months, 95% CI: 20.00-not estimable), but this was descriptive and did not reach statistical significance.

**Table 3:** Kaplan–Meier subgroup comparisons for recurrence-free survival and overall survival.

Subgroup/category	RFS events, n (%)	mRFS, months (95% CI)	Log-rank *P*	OS events, n (%)	mOS, months (95% CI)	Log-rank *P*
Gender			**.150**			**.438**
Male	26 (49.1)	16.87 (12.79-not estimable)		36 (67.9)	21.11 (17.95–32.98)	
Female	14 (35.0)	Not estimable (14.56-not estimable)		23 (57.5)	22.13 (19.04-not estimable)	
Age group			**.408**			**.101**
≤50 yr	20 (54.1)	16.87 (13.09-not estimable)		21 (56.8)	25.38 (19.04-not estimable)	
>50 yr	20 (35.7)	Not estimable (16.01-not estimable)		38 (67.9)	20.09 (18.64–26.56)	
Apatinib treatment			**.115**			**.027**
No	28 (36.8)	Not estimable (16.31-not estimable)		51 (67.1)	20.12 (19.04–25.38)	
Yes	12 (70.6)	16.87 (11.51-not estimable)		8 (47.1)	39.12 (20.12-not estimable)	
Bevacizumab treatment			**<.001**			**.499**
No	26 (33.8)	Not estimable (19.13-not estimable)		50 (64.9)	20.55 (19.04–35.87)	
Yes	14 (87.5)	8.22 (5.49-not estimable)		9 (56.2)	32.98 (17.95-not estimable)	
Unawareness			.643			.843
No	31 (44.9)	20.68 (14.56-not estimable)		45 (65.2)	21.11 (19.07–35.87)	
Yes	9 (37.5)	Not estimable (16.01-not estimable)		14 (58.3)	24.49 (14.50-not estimable)	
Full awareness			.323			.511
No	27 (39.1)	Not estimable (16.01-not estimable)		41 (59.4)	21.67 (19.07–39.12)	
Yes	13 (54.2)	16.87 (11.84-not estimable)		18 (75.0)	21.11 (17.49–39.91)	
Awareness of lesser severity			.495			.080
No	34 (46.6)	19.13 (14.30-not estimable)		45 (61.6)	23.74 (19.96–39.91)	
Yes	6 (30.0)	Not estimable (14.56-not estimable)		14 (70.0)	19.79 (13.81-not estimable)	
Midway awareness			.931			.075
No	28 (41.2)	20.68 (16.01-not estimable)		46 (67.6)	20.12 (18.41–24.49)	
Yes	12 (48.0)	24.99 (10.82-not estimable)		13 (52.0)	32.98 (19.96-not estimable)	
Disease awareness category			**.734**			**.167**
Unawareness	9 (37.5)	Not estimable (16.01-not estimable)		14 (58.3)	24.49 (14.50-not estimable)	
Full awareness	13 (54.2)	16.87 (11.84-not estimable)		18 (75.0)	21.11 (17.49–39.91)	
Awareness of lesser severity	6 (30.0)	Not estimable (14.56-not estimable)		14 (70.0)	19.79 (13.81-not estimable)	
Midway awareness	12 (48.0)	24.99 (10.82-not estimable)		13 (52.0)	32.98 (19.96-not estimable)	

*P*-values were calculated using the log-rank test. Median survival values are reported in months with 95% confidence intervals. “Not estimable” indicates that the median survival or upper confidence limit was not reached.

CI = confidence interval, mOS = median overall survival, mRFS = median recurrence-free survival, OS = overall survival, RFS = recurrence-free survival.

### 3.4. Prognostic factor analysis

After univariable and multivariable analyses, apatinib treatment was independently associated with OS (hazard ratio [HR] = 0.449, 95% CI: 0.211–0.957, *P* = .038), while bevacizumab was independently associated with RFS (HR = 4.359, 95% CI: 2.229–8.526). Disease awareness category was not significantly associated with either RFS or OS after adjustment for potential confounders (Table 4). Midway awareness showed a nonsignificant numerical pattern toward longer OS in both univariable (HR = 0.575, 95% CI: 0.310–1.067, *P* = .079) and multivariable analyses (HR = 0.631, 95% CI: 0.331–1.204, *P* = .163), and this finding should be interpreted as exploratory.

**Table 4:** Univariable and multivariable Cox regression analyses for recurrence-free survival and overall survival.

Outcome	Variable	Univariable HR (95% CI)	*P*	Multivariable HR (95% CI)	*P*
RFS	Age (>50 vs ≤50 yr)	0.768 (0.409–1.440)	.410	Not included	
	Gender (male vs female)	1.604 (0.837–3.073)	.155	Not included	
	Apatinib treatment (yes vs no)	1.720 (0.869–3.401)	.119	Not included	
	Bevacizumab treatment (yes vs no)	4.359 (2.229–8.526)	<.001	4.359 (2.229–8.526)	<.001
	Unawareness (yes vs no)	0.839 (0.398–1.768)	.644	Not included	
	Full awareness (yes vs no)	1.396 (0.718–2.715)	.325	Not included	
	Awareness of lesser severity (yes vs no)	0.739 (0.309–1.767)	.497	Not included	
	Midway awareness (yes vs no)	1.030 (0.522–2.035)	.931	Not included	
OS	Age (>50 vs ≤50 yr)	1.563 (0.912–2.679)	.104	Not included	
	Gender (male vs female)	1.230 (0.727–2.079)	.440	Not included	
	Apatinib treatment (yes vs no)	0.437 (0.205–0.930)	.032	0.449 (0.211–0.957)	.038
	Bevacizumab treatment (yes vs no)	0.783 (0.385–1.595)	.501	Not included	
	Unawareness (yes vs no)	0.941 (0.516–1.716)	.843	Not included	
	Full awareness (yes vs no)	1.204 (0.691–2.100)	.513	Not included	
	Awareness of lesser severity (yes vs no)	1.710 (0.931–3.143)	.084	1.393 (0.738–2.629)	.307
	Midway awareness (yes vs no)	0.575 (0.310–1.067)	.079	0.631 (0.331–1.204)	.163

Hazard ratios >1 indicate a higher hazard of recurrence or death, whereas hazard ratios <1 indicate a lower hazard. Variables with *P* < .1 in univariable analyses were included in the multivariable models. “Not included” indicates variables that did not meet the prespecified criterion for entry into the multivariable model.

CI = confidence interval, HR = hazard ratio, OS = overall survival, RFS = recurrence-free survival.

## 4. Discussion

Although data on disease awareness in relation to prognosis are available for other cancers,^[19-23]^ no such data were available for glioblastoma, which is the innovation of this single-center, retrospective cohort study. The main finding of the present study was that disease awareness category was not significantly associated with OS or RFS in patients with glioblastoma in China. The numerically longer OS observed in the midway awareness group should be interpreted as an underpowered, descriptive finding rather than evidence of a confirmed association. This study provides preliminary data in the field of disease awareness and prognosis in patients with glioblastoma, but larger prospective studies are needed before clinical implications can be drawn.

Such communication practice is rare in Western countries, but it is relatively common in China.^[10-12]^ The nonsignificant numerical pattern for midway awareness was observed for OS but not for RFS. One possible explanation is the lack of a clear temporal relationship between RFS and the timing of disclosure in patients who became aware during treatment. Some patients may have learned of their disease status after experiencing recurrence, which would weaken any causal interpretation for RFS. The OS pattern could also be due to the small sample size, which limits statistical power. Glioblastoma has a low incidence,^[5,6]^ resulting in a small sample size and attenuating statistical associations. Alternatively, disease awareness may be a weaker factor than established prognostic factors such as treatment. In the context of a small sample size and potential confounding, smaller effects are difficult to detect. Although stress, anxiety, and depression have been reported to influence the course of primary brain tumors,^[24]^ glioblastoma is highly aggressive, and the effect of awareness may be outweighed by tumor biology and treatment factors. Therefore, the present findings should be regarded as hypothesis-generating.

Full awareness of disease and prognosis can influence disease-related decisions, treatment adherence, lifestyle habits, emotions, and quality of life, including in patients with glioblastoma.^[29]^ In the present study, midway awareness was treated as a distinct category rather than as equivalent to full awareness because it referred to patients who learned the true diagnosis or severity after an initial period of limited awareness. This gradual process could, in theory, allow psychological preparation and promote treatment cooperation. Conversely, immediate full disclosure can be emotionally difficult for some patients if not accompanied by adequate support.^[27,31,32]^ Patients who remain unaware or are told a less severe explanation may have less direct involvement in medical decision-making, which could affect adherence and satisfaction with care. Nevertheless, because the current analyses did not show a statistically significant association between awareness category and OS or RFS, these explanations should be viewed as speculative. The findings do not support recommending any uniform disclosure strategy based on survival outcomes alone.

Psychological support remains an important component of cancer care.^[15,33,34]^ Such support can include cognitive behavioral therapy, pharmacologic treatment when indicated, and group therapy.^[35-37]^ The present study does not establish that disclosure level directly changes survival, and it should not be used as evidence to withhold or delay information from patients. Rather, it emphasizes that communication about diagnosis and prognosis in glioblastoma should be individualized, culturally sensitive, ethically appropriate, and supported by a careful assessment of each patient’s preferences and psychological readiness. Prospective studies that collect standardized measures of awareness, mood, quality of life, treatment adherence, and survival are needed to clarify whether and how disease awareness influences outcomes.

Nevertheless, this study has limitations that should be considered when interpreting the results. Its retrospective, single-center design is susceptible to selection bias and unmeasured confounding. Although every effort was made to include consecutive patients, those lost to follow-up could have introduced bias if their outcomes were systematically different from those who were followed. The small sample size, while understandable given the rarity of glioblastoma, limits the statistical power to detect weaker associations, such as the potential effect of disease awareness. Furthermore, the long enrollment period may have introduced biases due to evolving treatment standards. Disease awareness was retrospectively classified from clinical documentation and follow-up records rather than from a standardized prospective questionnaire; therefore, misclassification and subjectivity are possible. The timing of disclosure was also not uniformly recorded, limiting causal interpretation of the relationship between awareness and survival outcomes. While these constraints did not preclude a valuable preliminary investigation, they underscore the need for our findings to be validated in larger, prospective, multicenter studies.

## 5. Conclusion

Although disease awareness level was not significantly associated with OS or RFS in Chinese patients with glioblastoma, the midway awareness group showed a nonsignificant numerical pattern toward longer OS. This observation should be interpreted as exploratory and hypothesis-generating rather than evidence of a confirmed prognostic effect. Future large-scale, prospective, multicenter studies using standardized assessment of disease awareness are needed to clarify the relationship between disease awareness and glioblastoma prognosis.

## Author contributions

**Conceptualization:** Yanjiao Wu

**Data curation:** Yanjiao Wu, Xiaoying Xue, Shuying Ji, Lei Tian, Linlin Su

**Formal analysis:** Xiaoying Xue, Lei Tian, Linlin Su, Junling Liu

**Investigation:** Xiaoying Xue, Shuying Ji, Lei Tian

**Methodology:** Yanjiao Wu, Xiaoying Xue, Lei Tian, Xuetao Han

**Resources:** Wenyan Wang, Xuetao Han

**Software:** Wenyan Wang, Xuetao Han

**Visualization:** Shuying Ji, Wenyan Wang, Xuetao Han, Linlin Su

**Validation:** Wenyan Wang, Xuetao Han, Linlin Su

**Writing – original draft:** Yanjiao Wu, Xiaoying Xue, Shuying Ji, Wenyan Wang, Lei Tian, Xuetao Han, Linlin Su, Junling Liu

**Writing – review & editing:** Yanjiao Wu, Junling Liu
